# Implementing and Transitioning a Laboratory Quality Management System from ISO 15189:2012 to ISO 15189:2022: Experience from the Malawi-Liverpool Wellcome Research Programme, Blantyre

**DOI:** 10.12688/wellcomeopenres.25356.2

**Published:** 2026-04-17

**Authors:** Dumizulu Tembo, Joshua S Kaphika, Ajisa Ahmadu, Lovemore Alufandika, Vincent Kalilangwe, Christopher Kukacha, Dumisani Kaphika, Funny T Lipenga, Mirriam Machonjo, Sekani Manda, Mazuba Masina, Emmanuel C Mchoma, Alice C Mnyanga, Innocent M Moyo, Fatsani P Mutala, Patrick Mzumara, Doris Shani, George P Selemani, Brigitte Denis

**Affiliations:** 1 Malawi Liverpool Wellcome Research Programme, Blantyre, Malawi

**Keywords:** Quality management systems, ISO15189, Accreditation, Quality Assurance, Medical research

## Abstract

**Background:**

The implementation of a Quality Management System (QMS) in line with ISO 15189:2022 is essential for clinical and research laboratories striving to achieve technical competence and consistent delivery of valid results. This article presents the approach undertaken by Malawi Liverpool Wellcome Research Programme (MLW) laboratories to integrate and manage the QMS based on ISO 15189 and steps taken to successfully transition to the new version of the same standard.

**Methods:**

The implementation process involved several key steps, including mentorship, conducting an initial gap analysis, and implementing the quality management system. A quality officer was appointed to oversee the process, supported by leadership engagement and staff training to establish core competencies. Additional activities included the development of required documentation, participation in proficiency testing and Internal Quality Control (IQC) programmes, and the verification of test methods. Quality indicators were established to monitor performance. During the transition to the updated ISO 15189 standard, a second gap analysis was conducted, emphasising risk-based thinking and alignment with patient-centred requirements.

**Results:**

The outcomes demonstrate enhanced laboratory performance, improved documentation, and a stronger teamwork and culture of quality. This experience offers practical insights for laboratories seeking to implement ISO 15189 and underscores the importance of tailored implementation strategies that reflect the organizational context and laboratory scope.

**Conclusions:**

The successful implementation of a QMS demands a comprehensive, system-wide approach and robust teamwork to achieve established goals effectively. It necessitates ongoing periodic assessments and substantial support human and financial resources. Furthermore, continuous training and mentorship by qualified QMS professionals are essential to foster a culture of continuous improvement.

## Background

### Introduction

Many countries in sub-Saharan Africa (SSA) are gradually embracing advanced technologies and modern methodologies. However, this progress is significantly hindered by some of the lowest levels of government health spending per capita, ^1^ which hinder investment in essential research and development, infrastructure, ^2^ technology, collaboration, and workforce training. ^3^ Widespread poverty, high national debt, and fiscal instability further exacerbate the situation, ^4, 5^ forcing many governments to rely heavily on external aid, which is often inadequate to support large-scale technological initiatives. Additional barriers include weak management systems, the absence of robust quality assurance programs, ^6, 7^ and underperforming public institutions, bureaucratic inefficiencies and a lack of regulatory oversight. ^8^ further impede progress of the effective implementation of health innovations across the region.

When considering essential health packages, quality medical laboratory services are indispensable due to their critical role in patient diagnosis, treatment and care. However, such laboratories often lack the necessary resources and support, primarily due to a misunderstanding of their functions. ^9^ In SSA, quality assurance in medical laboratories is influenced by a combination of technical capabilities, quality management systems (QMS), and staff motivational factors, ^6, 10^ which include lack of internal quality control, a lack of resources in terms of infrastructure, equipment and technical expertise, ^11, 12^ power supply interruption, and frequent supply chain disruptions. ^13^

In medical research and clinical practice, producing reproducible, reliable, and accurate results is essential for high-quality patient care ^1^ and credible scientific evidence. Therefore, the output of medical laboratories is most trusted when supported by a robust QMS, such as that outlined in international standards such as ISO 15189. Since 2011, the Malawi Ministry of Health (MOH), together with laboratory implementing partners, have made tremendous national efforts in developing and implementing QMS through as the WHO-AFRO Stepwise Laboratory Quality Improvement Process Towards Accreditation (SLIPTA), the Strengthening Laboratory Management Toward Accreditation (SLMTA) program, and ISO 15189 standards. ^2^ As of 2025, Malawi has 11 medical laboratories accredited to ISO 15189, ^3^ distributed across seven districts out of the 27. This highlights both progress and remaining gaps in achieving widespread quality-assured medical laboratory services. While existing evidence suggests that successful implementation often depends on strong mentorship and collaborative efforts, ^2^ there is limited published evidence describing how medical laboratories in Malawi have successfully implemented and sustained QMS, particularly in alignment with ISO 15189 and the transition to the updated ISO 15189:2022 standard. This study aims to address this gap by sharing the experience of the Malawi-Liverpool-Wellcome (MLW) Research Programme in its journey towards ISO 15189 accreditation, with a particular focus on transitioning to ISO 15189:2022. 

It has been observed that 80–90% of correct diagnostic decisions are based on the quality of medical and diagnostic laboratory results. ^14^ Thus, reliable laboratory results are essential for effective patient care, disease management, public health surveillance, and research advancements. Without quality control measures, misdiagnoses can occur, leading to inappropriate treatments, wasted expensive drug treatments, ^6^ worsening health conditions, and inaccurate public health information. ^9, 15, 16^ Additionally, over-treatment and overuse of antibiotics for inappropriate clinical circumstances is inevitable, leading to the emergence of drug-resistant microorganisms. ^17^

In today’s landscape, competition serves as a catalyst for enhancing service quality. To deliver high-quality services, it is imperative to ensure the availability of adequate resources and the implementation of comprehensive quality assurance systems. Overcoming persistent barriers demands strategic investments, targeted policy reforms, and strengthened collaboration among governments. ^10, 18, 19^ Ultimately, the integration of these strategies into routine practice is essential for safeguarding public health and achieving sustainable improvements. ^20^ In recent years, the establishment of the SLIPTA, the development of the SLMTA training program, and the launch of the African Society for Laboratory Medicine (ASLM) by the national and international partners have played a significant role to enhance sustainable laboratory capacities across Africa. These initiatives form the backbone for advancing national health laboratory infrastructure, capacity development, and quality improvement. Thus, while the SLIPTA and SLMTA offer the necessary pathways and support for laboratories to achieve and sustain accreditation through effective quality management practices, ISO 15189 sets the quality benchmark.

### What is ISO15189

The International Organization for Standardization (ISO) standard for medical laboratory accreditation, ISO 15189, specifies the requirements for quality and technical competence in medical laboratories, and management. Since its initial publication, this standard has undergone several revisions to remain relevant and effective. The latest version, published in 2022, includes technical updates and replaces ISO 15189:2012. It emphasizes risk management, patient-centred care, and the integration of new technologies in laboratory practices. Achieving accreditation under ISO 15189 is a measure of a laboratory’s quality and competency, ensuring accurate, reliable, and timely results in a cost-effective manner.

### Malawi-Liverpool Wellcome programme

The Malawi Liverpool Wellcome Programme (MLW), based in Blantyre, Malawi, was established in 1995 and is widely recognized as a leading collaborative research institution both locally and internationally for its dedication to conducting excellent research that benefits health and trains the next generation of researchers. With a strong focus on infectious diseases, MLW plays a critical role in public health by supporting evidence-based decision-making and healthcare policies in Malawi and beyond. The MLW diagnostic laboratory achieved ISO 15189:2012 accreditation in 2021, reflecting its commitment to conducting quality research. Recently, the MLW laboratory successfully transitioned to the 2022 version of ISO 15189, ensuring conformance to the latest laboratory standards. The laboratory is committed to providing high-quality laboratory support and diagnostic services at Queen Elizabeth Central Hospital, the largest referral hospital in Malawi.

The aim of this article is to highlight experiences from the MLW journey in ISO 15189 accreditation with an emphasis on transitioning to ISO 15189:2022. We describe the key steps taken, challenges encountered, and lessons learned, with the goal of providing practical insights to support other laboratories undergoing similar processes, as shared experiences can offer valuable insights, reduce implementation barriers, and promote best practices. MLW’s drive toward ISO accreditation stems from the need to strengthen laboratory QMS to ensure consistency, reliability, and compliance with global best practices. Accreditation aligns with MLW’s mission of conducting high-impact research and providing laboratory services that meet the highest standards. Furthermore, it enhances collaboration with international partners, supports regulatory compliance, and facilitates the integration of research findings into national and global health policies. ^21^

## Methodology

### Ethical considerations

This was a descriptive quality improvement study, not requiring ethical review as all information presented in the manuscript was derived exclusively from routine laboratory QMS operations, such as internal audits, document control, and process improvement activities. These activities were part of standard laboratory management practices and did not include identifiable patient information or clinical samples. The requirement for informed consent or assent was not applicable as human subjects were not included at any stage of this work.

### Gap analysis

In 2016, the laboratory engaged UMOYO Laboratory Consultants to support the development and implementation of its quality management system. To establish the status of the QMS of the laboratory and define a road map towards accreditation, a baseline assessment was conducted using the World Health Organization Regional Office for Africa (WHO/AFRO) Strengthening Laboratory Quality Improvement Process Towards Accreditation (SLIPTA) version 2:2015 to evaluate the laboratory’s QMS from 23 to 25 May 2016 based on the 12 quality System essentials of i) documents and records, ii) management reviews, iii) organisation and personnel, iv) customer service and client management, v) equipment, vi) evaluation and audits, vii) purchasing and inventory, viii) process control, ix) information management, x) identification of non-conformities, corrective and preventive action, xi) occurrence/incident management and process improvement and xii) facilities and safety.

UMOYO Consultants also delivered ISO15189:2012 management system training, which led the groundwork for the development of robust QMS practices. In addition, MiChem Dynamics Consultants were engaged in 2017 to provide training in internal auditing based on the ISO15189:2012, as a groundwork for the development of robust quality management practice. Overall, the identification of non-conforming areas was accomplished not only by using the SLIPTA checklist, but also another checklist based on ISO 15189:2012 standard. Both audit checklist questions covered both technical and management areas. The audit finding also guided the development of an action plan containing the details of observations, recommendations and activities to be actioned by the lab.

### Transitioning from ISO 15189:2012 to ISO 15189:2022

The transition from ISO 15189:2012 to ISO 15189:2022 was implemented through a structured and strategic approach. In 2024, a mentor from QualExpect Consult was engaged to support the process, delivering targeted training on ISO 15189:2022 management systems, risk management, and internal auditing. To assess readiness, a comprehensive gap analysis was conducted, guided by the ISO 15189:2022 Transition Document (SADCAS TR 29 Issue No.1, Approved 2023-02-21) issued by the Southern African Development Community Accreditation Service (SADCAS), the designated accreditation body. To ensure full compliance with the updated standard, a detailed transition plan was developed using SADCAS F 134 (d) Issue 1, dated 2023-11-18. It demonstrated our thorough evaluation of ISO 15189:2022 and its implications for our QMS. The transition plan specified key actions required for compliance, timelines, assigned responsibilities for each action, and monitoring for completion, was formally submitted to SADCAS on 31 December 2023. A key milestone in this process was the SADCAS assessment visit on 31 August 2024, conducted to verify the implementation of the planned actions.

### Implementation of the quality management system

The implementation of the QMS commenced with the presentation of the concept to senior management to secure their approval. This initial step was crucial in gaining the necessary support and resources for the project. Following approval, there was a significant restructuring of roles within the laboratory management team. The roles of the lab manager and assistant lab manager were redefined to align with the requirements of the ISO standard. A Quality Assurance Manager (QAM) was appointed, and the roles of senior technicians were redefined to include leadership responsibilities within the QMS framework. Subsequently, a series of training sessions were organized for the management team. These sessions were designed to provide comprehensive knowledge of the ISO 15189 standards and the specific requirements for QMS implementation. Following the management training, awareness meetings were conducted for the entire staff.

During the transition phase, the QMS had been in operation for six years, and the staff were already familiar with the general workflows involved in its establishment and maintenance. However, with the introduction of the new ISO15189:2022 standard, additional training was identified as necessary. This training focused on understanding the significant changes introduced in the new standard, with particular emphasis on risk-based thinking, requirements for point-of-care-testing, competence requirements and the need for ongoing development, and the performance monitoring and continual improvement using the plan-do-check-act (PDCA) cycle. The risk management training aimed to equip staff with the skills to identify, assess, and mitigate potential risks within the laboratory processes. Internal auditing training was also provided to ensure that the laboratory could conduct thorough and effective audits to maintain compliance with the ISO 15189:2022 standards.

### Document development

Although there was evidence of the implementation of the QMS through the documents and records generated, the existing laboratory policies and procedures required formalisation. The Quality Manual was revised to include missing elements, a list of other mandatory managerial Standard Operating Procedures (SOPs) listed in Table 1, based on their general applicability and their role in quality control were also developed. The documents were reviewed, approved, and disseminated to all staff. Furthermore, the laboratory identified additional areas of focus, including establishing a routine review of quality records at the department level, conducting annual management reviews, developing systems for assessing technical competency among staff, soliciting feedback from staff and clients on laboratory services, validating and verifying equipment, providing internal audit training, and selecting quality indicators.

**Table 1.** Laboratory quality management procedures.

	General	Quality Control
**1**	Contingency plan	Autoclave efficiency validation
**2**	Corrective actions	Competency assessments
**3**	Equipment management	Continual improvement
**4**	Evaluation of Suppliers	Creation and maintenance of training file for laboratory staff
**5**	External Quality Control Specimens	Document control
**6**	External services and supplier	Internal audits
**7**	Handwashing	Key Performance Indicators Management
**8**	Inventory management	Management review
**9**	Personnel management	Operations and maintenance of safety cabinet
**10**	Record control	Preventative Maintenance of Laboratory Equipment
**11**	Sample disposal and retention	Quality Assurance
**12**	Sample handling and transportation	Resolution of complaints
**13**	Service agreements	Risk management procedure
**14**	Specimen shipping	Temperature Monitoring
**15**	Spillage handling	
**16**	Staff and client service management	
**17**	Verification of methods	
**18**	Waste disposal	

To ensure a successful transition from ISO 15189:2012 to ISO 15189:2022, initially, the laboratory management and staff were committed to the transition process as their involvement was crucial for success. The next step involved aligning the laboratory’s quality objectives with the broader organisational strategy. To support this alignment, all quality procedures were thoroughly revised; including the Quality Manual, corrective action procedure, internal audit procedure, and risk management procedure, to ensure they incorporated risk-based thinking and conformed to the updated standards, with a strong emphasis on patient care. Additionally, the impartiality section was updated, and the sample transportation requirements were revised to better reflect the new structure and terminology.

### Staff training and competency assessment

The mandatory and relevant training programs were identified to enhance knowledge, skills, and competencies while fostering positive attitude changes within the team. This included ISO15189 training for a diverse range of staff members, including laboratory managers, QAM, laboratory technicians, and support personnel such as laboratory administrators and archivists. The subjects of training sessions, the number of staff trained, and the dates of the training are listed in Table 2. To promote strong teamwork and a cohesive understanding of the management systems. it was essential for all staff to undergo training simultaneously. The training primarily took place through workshops and coaching sessions, facilitated by a mentor. Each training session concluded with quizzes to evaluate participants’ understanding of the procedures discussed, with a minimum passing score of 70%. Those who did not achieve this score were awarded a certificate of attendance. An SOP for competence assessment was established, assigning the responsibility for training on technical procedures to the senior technician or quality officer. Competence was assessed through observational methods or successful analysis of known samples, internal controls, or External Quality Assessments (EQA). The QAM or Senior Technician was tasked with ensuring that all competence assessment forms were completed and appropriately signed.

**Table 2.** Training sessions given to staff.

*Training*	*No. of Staff trained*	* Training dates*
Introduction to Quality management system	48	1 ^st^ August 2016
Review of the ISO15189:2012 standard	48	1- 6 August 2016
Review of the ISO15189:2022 standard	40	9 – 11 November 2023
Internal audit training course based on ISO19011:2018	18	3 – 7 ^th^ June 2024
Risk management training based on ISO22367:2020	20	20 – 22 May 2024

### Method verification

The laboratory developed detailed procedures to guide staff to distinguish and perform the verification of technical methods. Upon completion of all verification experiments, raw data and results were compiled and retained indefinitely. A summary of verification, signed by the competent qualified authorized staff member, confirms that the verification process has been reviewed and approved. Regular verification of documented performance is conducted through Internal Quality Control (IQC) and EQA methods.

Verification involved using known traceable sample including IQC and EQA samples to confirm Manufacturer performance characteristics. Competent staff members performed tests under verification, comparing results to expected outcomes. Results meeting established criteria or manufacturer claims provided evidence of method suitability, while those failing to meet criteria indicated method inadequacy for patient testing. Depending on the type of method, a number of analyses were employed; accuracy, specificity, sensitivity, linearity, and precision were determined. Precision was assessed by calculating coefficients of variation (%CV), and accuracy was determined to obtain confidence intervals (CI). Specificity evaluated the assay’s ability to correctly identify true negative samples, while uncertainty of measurement characterised result dispersion. Precision measured the agreement between replicate measurements, and linearity assessed the method’s ability to provide proportional test results. Accuracy was evaluated based on the agreement between test results and an accepted reference value obtained from a large series of tests.

### Internal and External Quality Assessments

The laboratory procured IQC samples and enrolled all procedures for patient sample processing in an appropriate in an EQA program. To ensure effective implementation of both the IQC and EQA plans, a documented procedure was established as guidance. Part of the laboratory staff’s competence was to evaluate the results of IQC and EQA samples before reporting patient samples. Initially, EQA average monthly performances was set to 80% and revised to 85% during the transitioning period. When this score was not achieved, it was documented as a Non-Conformance (NC), which required root cause analysis and implementation of Corrective Actions (CA).

### Quality Indicators

Quality Indicators (QI) were defined as any measure of a system where data collected over a specified period are analysed to determine the improvement of the established system. The QI included: turnaround time, ensuring that 80% of all released patient reports are released within the agreed timeframes, EQA achieve a minimum average performance of ≥80% monthly, zero days of unplanned service interruption, less than 1% specimen rejection per month, zero days of unplanned equipment downtime, no stockouts, less than 10% Cerebral Spinal Fluid (CSF) contamination rates, and blood culture contamination rates less than 15%. QIs were reviewed by the QAM and the Laboratory Manager. Deviations from the set targets resulted in an NC and prompted CAs based on the QMS established under ISO 15189:2012. The QIs were reviewed semi-annually, led by the QAM. In preparation of the transitioning process, the Turn Around Time (TAT) and EQA performance targets were revised to 85% effective August 2024. Also, management agreed to stop monitoring reagent stockouts and instead monitor service interruptions only. Equipment downtime and reagent stockout were no longer monitored separately but included under service interruptions.

## Results

### Baseline assessment

**Figure 1.**
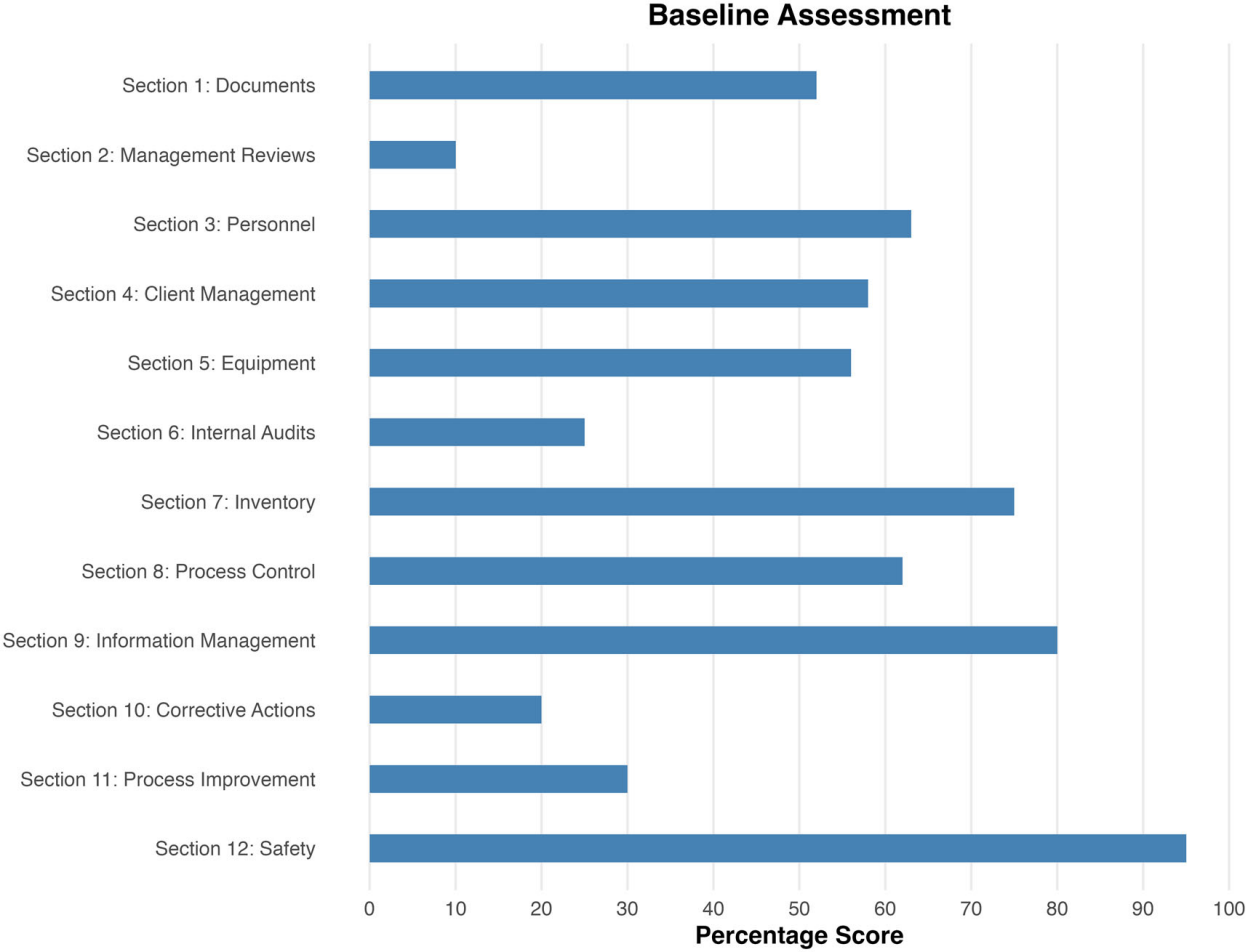
Baseline assessments. WHO AFRO SLIPTA Checklist for Malawi Liverpool Wellcome Research Program.

The laboratory achieved a score of 166 out of a possible 275 (60%), which corresponds to one star on the WHO/AFRO SLIPTA checklist, a system that evaluates laboratory performance on a scale of 0 to 5 stars. Figure 1 illustrates the laboratory’s performance across the 12 sections of this checklist. Notably, the laboratory management demonstrated a strong commitment to QMS implementation, as evidenced by high scores in three key areas: inventory management, information management, and safety. However, the laboratory did not perform well in several critical aspects, scoring below 50% in management reviews, internal audits, CAs, and process improvements. These areas are vital for ongoing continuous improvement in the laboratory, serving as foundational elements for identifying opportunities for enhancement.

During the training and review of the ISO15189:2012 standard, all system procedures required by the laboratory were identified and developed. More than 95% of the training time was dedicated to the reviewing of the standard. As a key adult learning principle, the sessions used plenary discussions as a means of learning to ensure participation and sharing of experience from the trainees. Considering that quality and technical records are essential evidence of implementing of QMS, the laboratory started documenting all their QMS activities to create a trail of evidence.

### Periodic assessments and accreditation

Between October 2020 and July 2025, the laboratory underwent six assessments, initially under ISO 15189:2012 and later under ISO 15189:2022. Table 3 provides a summary for all the assessments and their recommended areas of improvement, A total number of 22 NCs (19 major and 3 minor) arose at the initial assessment based on the ISO15189:2012, with recommendations for improvements focused on adherence to procedures, reporting of Laboratory Information Management Systems (LIMS)-generated results, and EQA performance. The first and second periodic assessments between 2021 and 2022 reported 17 and 18 major NCs respectively, revealing recurring issues such as adherence to procedures, non-conformity management, and documentation practices. The 2023 assessment identified 20 major and 4 minor NCs, including re-raised NCs that indicated that some CAs were only partially resolved. The recommendations emphasised on staff training, record and inventory management, and QMS ownership.

**Table 3.** A summary of the assessments over a five-year period cycle and the recommended areas for improvements

*Assessment Type*	*Date*	*Mode*	*Standard*	*Non-Conformances *		*Recommended Areas for Improvement*
				* major*	* minor*	
Initial Accreditation Assessment	5 – 6 October 2020	Remote	ISO 15189:2012	*19*	*3*	- Adherence to documented procedures - LIMS-generated result reports - Strengthen EQA performance
First Periodic Assessment and Scope Extension	26 August 2021	Remote	ISO 15189:2012	*17*	*1*	- Adherence to documented procedures - Non-conformity management and inventory control - Manual EQA result documentation
Second Periodic Assessment	6 September 2022	Physical	ISO 15189:2012	*18*	*1*	- Monitoring and strengthening of CA, especially where EQA performance is poor - Staff familiarity with quality documentation - Improve handwritten amendments - Timeliness on NC resolution
Third Periodic Assessment	30 August 2023	Hybrid (Physical and Remote)	ISO 15189:2012	*20*	*4*	- Staff training and QMS ownership - Ethical conduct arrangements - Adherence to documented procedures - Inventory and record management
Fourth Periodic Assessment	5 – 6 August 2024	Physical	ISO 15189:2022	*29*	*0*	- Strengthen RCA - Preventing recurrence of non-conformities
Fifth Periodic Assessment and Scope Extension	23 – 24 July 2025	Physical	ISO 15189:2022	*40*	*1*	- Comprehensive RCA and monitoring of the effectiveness of CA for poor EQA performance must include determining clinical significance - Comprehensive and rigorous risk management - Understanding and implementation of verification methods - Adherence to documented procedure

Abbreviations: CA: corrective action; EQA: External Quality Assurance; NC: Non-conformance, LIMS: Laboratory Information Management Systems; RCA: Root Cause Analysis; QMS: Quality Management Systems

### The transitioning to ISO15189:2022

A comprehensive gap analysis against the ISO 15189:2022 standard was conducted on 18 November 2024. During the transition assessment, the laboratory was found to have 29 major non-conformances, reflecting significant challenges in adapting to the updated ISO 15189:2022 requirements. The areas for improvement focused on strengthening root cause analysis and preventing recurrence. The fifth periodic assessment in 2025, six months post-transition, recorded 40 major and 1 minor non-conformance, with recommended improvements including comprehensive root cause analysis, monitoring of CAs, rigorous risk management, and complete implementation of verification of methods. This marked the highest number of findings and concluded the periodic assessment cycle.

### MLW laboratories performance based on established Quality Indicators

**Figure 2.**
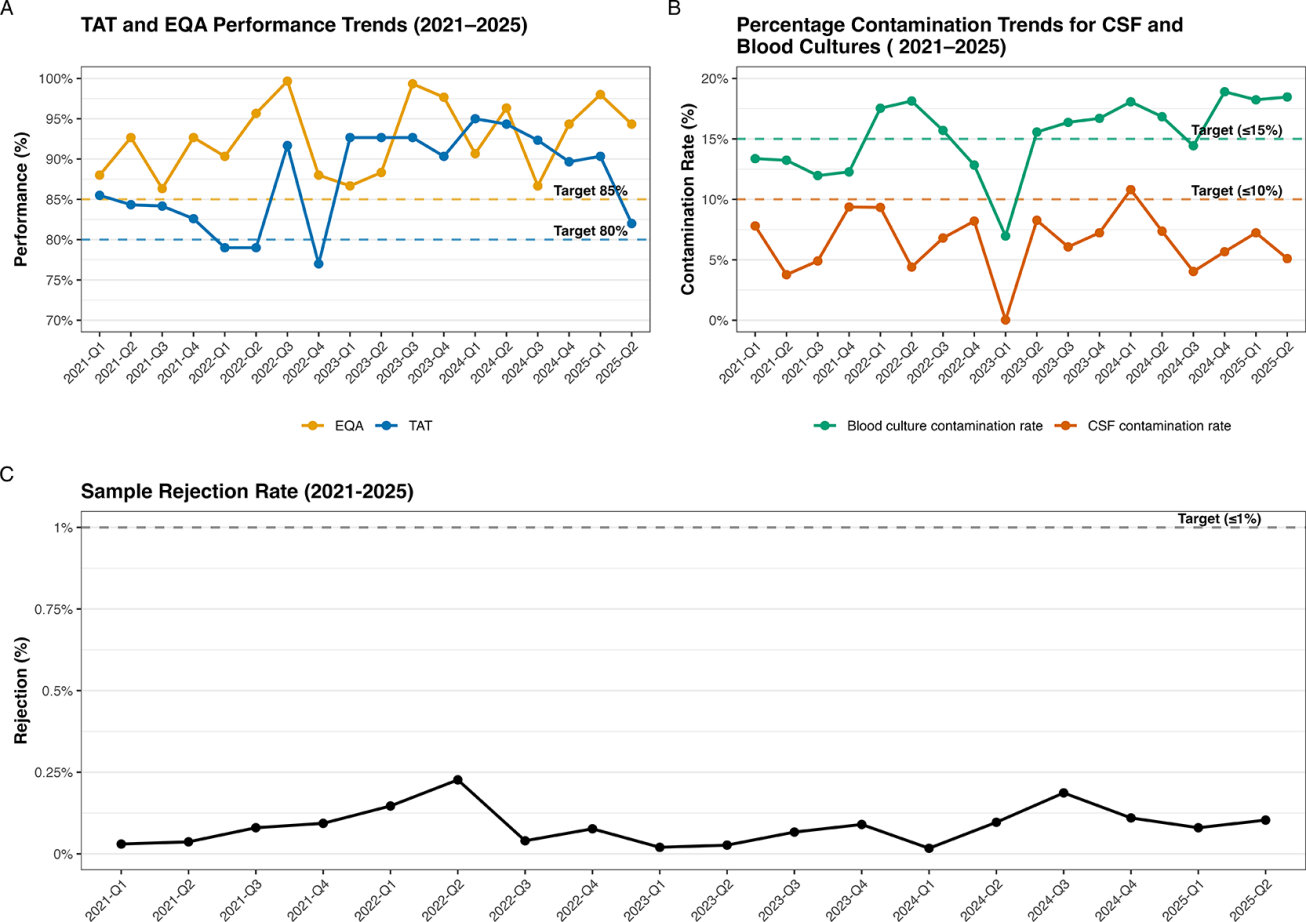
Quarterly performance of key quality indicators following the implementation of ISO15189:2012 and ISO15189:2022. ( **A**) Turnaround time (TAT) and external quality assessments (EQA) performance; ( **B**) Blood culture and cerebral spinal fluid contamination rates; and ( **C**) Sample rejection rates. ISO15189:2022 was implemented starting July, the third quarter of 2024 (2024-Q3), accompanied by increased target for TAT and EQA performance to 85%. During this period, the metric for service interruptions was also increase to include equipment downtime and stockouts.

Following the development of QIs, these were reviewed and discussed during the laboratory’s annual management review meeting. The minutes of this discussion were subsequently circulated to all laboratory staff to ensure transparency and collective ownership of performance improvement initiatives. CAs were implemented for all KPIs that did not meet their predefined targets, with prioritisation based on the level of risk posed to patient care. Performance data during ISO15189:2012 between 2021 and the second quarter of 2024 indicated that most KPIs met their expected targets, strong TAT consistently above the 80% target, EQA scores frequently above 90% ( Figure 2A), and minimal stock-outs or equipment downtime ( Table 4). Overall, the laboratory maintained strong performance with TAT averaging 87.8%, EQA 92.0%, and rare service interruptions (0.15/quarter) ( Table 5). Specimen rejection (averaging 0.19%) and CSF contamination rates (averaging 6.51%) were generally well within goals, though occasional spikes occurred. The main persistent challenge was blood culture contamination, which often hovered around or slightly above the 15% limit ( Figure 2B). Following implementation of the revised standard, accompanied by higher targets for TAT and EQA raised to 85%, and a broader service interruption metric, performance remained high. TAT and EQA consistently met or exceeded the new thresholds (averaging 86.2% and 96.2% respectively), service interruptions were rare (average 0.34/quarter) ( Table 5), and sample rejection ( Figure 2C) and CSF contamination rates ( Figure 2B) stayed within acceptable limits (averaging 0.09% and 6.17% respectively. However, blood culture contamination continued to exceed the 15% benchmark, highlighting an ongoing area for quality improvement under the updated system.

**Table 4.** Service interruptions, stock-outs and equipment downtime post ISO15189 implementation.

INDICATOR	GOAL	2021				2022				2023				2024				2025	
		Q1	Q2	Q3	Q4	Q1	Q2	Q3	Q4	Q1	Q2	Q3	Q4	Q1	Q2	Q3	Q4	Q1	Q2
**Service Interruptions**	None	1.0	0.0	0.0	0.0	0.0	0.0	0.0	0.0	0.3	0.0	0.0	0.0	1.0	0.0	0.0	0.0	0.0	0.7
**Stock Outs**	None	3.7	2.0	3.5	0.0	0.3	0.0	1.0	0.0	0.3	0.0	0.0	0.0	0.0	0.0				
**Equipment Down Time**	< 1 day/ month	0.0	0.7	1.0	0.7	0.0	0.3	0.0	0.0	0.3	0.0	0.0	0.0	0.3	0.0				

First quarter (Q1) starts January of each year. The grey area indicates an increase metric for service interruptions, which included equipment downtime and stockout starting third quarter (Q3) of July 2024.

**Table 5.** Comparison of KPI performance before and after ISO 15189:2022 implementation.

Indicator	ISO 15189:2012 Avg (2021 Q1 – 2024 Q2)	ISO 15189:2022 Avg (2024 Q3 – 2025 Q2)	Target/Benchmark
**Turnaround Time (%)**	87.8	86.2	80% (raised to 85% post-ISO15189:2022)
**External Quality Assessment (%)**	92.0	96.2	80% (raised to 85% post-ISO15189:2022)
**Service Interruptions (events/quarter)**	0.15	0.34 *	None
**Stock-outs (events/quarter)**	0.77	N/A ^†^	None
**Equipment Down Time (days/month)**	0.24	N/A ^†^	<1 day/month
**Specimens Rejected (%)**	0.19	0.09	<1%
**Cerebral Spinal Fluid Contamination (%)**	6.51	6.17	<10%
**Blood Culture Contamination (%)**	14.89	18.35	<15%

* Service interruptions post-ISO15189:2022 include stock-outs and equipment downtime.

^†^ Stock-outs and equipment downtime were no longer tracked separately after July 2024.

### Accreditation

Following 2 years of intensive preparation, the laboratory achieved ISO15189:2012 accreditation on 16 February 2021, after satisfactorily addressing all NCs. The accreditation was granted by the SADCAS Accreditation Approval Committee under registration number MED061. Accreditation was successfully awarded for tests performed across six major laboratory disciplines: Bacteriology, Mycobacteriology Parasitology, Chemistry, Serology, and Haematology. This milestone marked a significant enhancement in the laboratory’s QMS and technical competence, affirming its commitment to international standards. To transition to the 2022 version of the standard, the laboratory took a comprehensive quality improvement roadmap, beginning with a gap analysis conducted on 18 November 2023. The formal assessment took place on 6 August 2024, and because of this proactive approach, the laboratory achieved accreditation under ISO 15189:2022 on 7 January 2025. The laboratory has expanded its scope of accreditation by adding additional tests under the chemistry discipline and incorporating molecular biology into its existing six accredited scopes. As a result, a total of 67 examination procedures are now accredited under ISO 15189:2022. This experience underscored the importance of early planning, comprehensive staff engagement, and robust internal audit mechanisms when navigating standard transitions of this magnitude.

## Discussion

The implementation of ISO 15189:2012 was successfully completed within two years, marking a significant milestone in the laboratory’s journey toward accreditation. Notable progresses were observed during baseline, particularly in the areas of facilities and safety and information management. These achievements highlight the effectiveness of the SLMTA approach in building capacity and enabling laboratories to progressively improve quality services to meet accreditation standards. Despite these gains, progress in implementing certain requirements in the standard was slow. This was largely attributed to the laboratory staff’s low moral due to unfamiliarity with the standard and a weak internal audit system to sufficiently identify the gaps in the system. Only one internal audit was conducted in October 2015, and at the time, the laboratory lacked trained internal auditors. While corrective actions were being recorded, other key information such as proper monitoring of the effectiveness of corrective actions, was not adequately captured.

The progressive investment in staff trainings conducted on-site were found to be effective in addressing such concerns by enhancing skills, clarifying roles and boosting employees’ confidence. Early foundational training in the ISO15189 standard, supplemented by more specialized training in internal auditing and risk management ensured the availability of advanced competencies in alignment with the new requirements for the sustainability of the accreditation. In support, some studies demonstrate that hand-on training accelerates QMS adoption in medical laboratories. ^22– 24^ The laboratory management, comprising of the senior technicians and the laboratory managers also adopted an open-door policy, combined with regular staff meetings and the provision of adequate resources, to foster a collaborative environment conducive to continuous improvement. This participatory management style also encouraged staff to proactively identify and close quality gaps. The laboratory appointed a quality officer who was dedicated and had the leadership skills, to ensure the adherence to the established systems. The appointment of a quality office offers a strength in the laboratory management and assist the implementation process to move faster and efficiently. ^25, 26^ Overall, staff commitment and strong teamwork contributed significantly to the laboratory’s improved performance in the eventual success of the QMS. The laboratory also received strong backing from the senior management team, which according to, ^27^ is a key success factor in the implementation of QMS in developing countries, to ensure the provision of essential financial support and organisational condition.

Since the implementation of the ISO15189-based QMS, the laboratory has also provided regular objective assessments through internal and external assessment ensuring continuous improvements in operations. Across six assessments conducted between 2020 and 2025, the laboratory demonstrated progress in maintaining accreditation. However, NCs persisted through the accreditation cycle with fluctuations in both major and minor findings, particularly in procedural adherence, NC management, and documentation practices, highlighting partially resolved corrective actions. These challenges were further compounded by competing priorities, and the complexity of maintaining routine laboratory operation while undergoing a major system overhaul. This may allude to residual challenges such as resistance from staff, stemming from the cultural and procedural shifts required during the accreditation process. Similar issues have been, described from other studies conducted in Kenya. ^26^ and Rwanda. ^28^ However, it also reflects an increase in system sensitivity and more rigorous assessment against stringent requirements in root cause analysis, CA effectiveness, particularly following the transition to ISO15189:2022, which was accompanied by a sharp increase in NCs. Despite these obstacles, corrective and preventive actions emphasizing addressing the newly introduced requirements were implemented and monitored. The efforts ensured that the management system documentation was not only compliant but also functionally aligned with day-to-day operations, thus fostering a culture of continuous improvement and regulatory alignment. As the QMS matures, it is anticipated that the frequency of such NCs will decrease. Nonetheless, it is acknowledged that sustaining a QMS requires not only structural system, but also staff require time to adapt to the new systems and internalise a quality-focused mindset.

The transition to ISO 15189:2022 was relatively seamless, suggesting that the laboratory’s existing foundational structures and quality management processes were robust. Staff demonstrated strong commitment and teamwork throughout the process, willingly providing the necessary documentation and actively participating in gap analyses to guide quality improvements. The full transition was completed within 14 months from the initial gap analysis. Regular mentorship from experienced professionals played a critical role in promoting accountability and fostering a collaborative environment. The positive impact of mentorship on the implementation of QMS has also been documented in other regions, including Malawi, ^29^ Botswana, ^30^ Kenya, ^31^ and Zimbabwe. ^32^ Furthermore, studies by Ntshambiwa *et al..* ^33^ and Makokha *et al..* ^34^ found that laboratories with active mentorship programs were significantly more likely to achieve and sustain accreditation, reinforcing the value of structured support in laboratory quality improvement efforts.

The laboratory has recorded notable improvements in QIs. Overall, the lab experienced minimal service interruptions throughout the accreditation cycle, indicating relatively stable laboratory operations. The slight increase of 0.7 observed in the second quarter of 2025 was due to the chemistry analyser being down, including at the back up lab. This period also coincides with missing the TAT target, highlighting the direct relationship between instrument downtime and delays in releasing results. ^4^ Maintaining minimal equipment downtime and service interruption is critical ensuring continuously of diagnostic services and supporting timely clinical decision-making. ^5^ These observations also emphasise the need for strong collaboration between laboratories, in sharing equipment, capacity, and technical support. Strengthened collaboration helps mitigate risks, reduce TAT delays, and maintain service continuity when workload increases or when during system failure. ^6^ Despite the temporary disruption, overall, TAT remained above the target benchmark, reflecting well-optimised workflows.

EQA performance before and after the transition to ISO15189:2022 remained largely positive, consistently above the 85% pass target. This indicates enhanced analytical accuracy and reliability of laboratory results. Similarly, specimen rejection rate remained below target at about 0.2%, suggesting a continued improvement in pre-analytical processes and sample handling. In resource-limited setting, lower sample rejection rates are important as they reduce the need for repeat sampling, minimise delay in patient care and optimise the use of resource. ^7^^,^^8^

CSF contamination rates consistently remain below the acceptable limit of 10% while blood contamination rate blood consistently out of the acceptable target of 15%. According the the international gold standard by the Clinical and Laboratory Standard Institute and College of American pathologist clinically acceptable rate for blood cultures at ≤ 3. However, persistent challenges remain, concerning elevated contamination rates in blood culture samples. Evidence indicates that samples collected under direct laboratory oversight exhibit significantly lower contamination rates than those obtained from external sources. To address this, the laboratory has emphasised the need for sustained and targeted engagement with relevant stakeholders, especially the clinical teams involved in sample collection. This includes structured training programs, collaborative initiatives, and continuous dialogue to reinforce best practices. Regular monitoring and evaluation of these interventions are essential to assess their effectiveness and ensure compliance with established protocols. The overarching goal is to reduce contamination rates in both CSF and blood cultures to a single digit percentage that is both achievable and clinical acceptable. Given the potential implications for diagnostic accuracy and patient outcomes, the elevated contamination rate has been identified as a significant risk and has been formally documented in the laboratory’s risk register and is subject to ongoing surveillance as part of the laboratory’s risk management.

## Conclusions

The findings from this study demonstrate that the implementation and continuous strengthening of our QMS is aligned with the ISO15189 requirements. Based on our experience, the successful implementation and maintenance of a QMS requires a comprehensive, system-wide approach supported by collaborative teamwork. Regular assessments involving all stakeholders, along with strong human and financial resource backing, are essential. Key components such as internal audits, periodic objective reviews, a dedicated quality office, and committed laboratory management are critical for sustaining QMS effectiveness. Furthermore, continuous training, staff motivation, and mentorship by qualified QMS professionals help foster a culture of quality and innovation. By embracing these principles, organizations can achieve not only regulatory compliance but also continuous improvement and long-term operational excellence.

## Data Availability

Figshare: Implementing QMS based on ISO15189 at MLW Laboratories_Data collected and Analysed. https://doi.org/10.6084/m9.figshare.30999691. ^35^ The project contains the following underlying data: Implementing QMS based on ISO15189 at MLW Laboratories_Data collected and analysed.xlsx. The dataset includes training conducted, non-conformances identified, key performance indicators tracked, and standard operating procedures developed and implemented. Implementing QMS based on ISO15189 at MLW Laboratories_Data collected and Analyse dataset. The analysis was conducted using R, an open-source statistical software. Data are available under the terms of the Creative Commons Attribution 4.0 International license (CC-BY 4.0).

## References

[1] Global Burden of Disease Health Financing Collaborator Network: Past, present, and future of global health financing: a review of development assistance, government, out-of-pocket, and other private spending on health for 195 countries, 1995–2050. *Lancet.* 2019;393(10187):2233–2260. 10.1016/S0140-6736(19)30841-4 31030984 PMC6548764

[2] Beyene E Bedemo A Gebremeskel A : Determinants of digital technology development in sub-Saharan African countries: evidence from panel data analysis. *Energy Inform.* 2024;7(1): 21. 10.1186/s42162-024-00324-4

[3] Ngoma C Phiri WKB Chidzaye R : Enhancing public health through multi-stakeholder collaboration in Africa. *Ann Med Surg (Lond).* 2024;86(10):5672–5675. 10.1097/MS9.0000000000002532 39359784 PMC11444543

[4] Anakpo G Xhate Z Mishi S : The policies, practices, and challenges of digital financial inclusion for sustainable development: the case of the developing economy. *FinTech.* 2023;2(2):327–343. 10.3390/fintech2020019

[5] Comelli F Kovacs P Villavicencio JJM : Navigating fiscal challenges in Sub-Saharan Africa: resilient strategies and credible anchors in turbulent waters. *Departmental Papers.* 2023;2023(007):74. 10.5089/9798400230349.087

[6] Mesfin EA Taye B Belay G : Factors affecting quality of laboratory services in public and private health facilities in Addis Ababa, Ethiopia. *EJIFCC.* 2017;28(3):205–223. 29075171 PMC5655637

[7] Schmidt AM Abad G Brearey S : Managing regulatory issues arising from new diagnostic technologies: high throughput sequencing as a case study. *CABI Agric Biosci.* 2025;6(1): 0024. 10.1079/ab.2025.0024

[8] Olmsted SS Moore M Meili RC : Strengthening laboratory systems in resource-limited settings. *Am J Clin Pathol.* 2010;134(3):374–380. 10.1309/AJCPDQOSB7QR5GLR 20716792

[9] Christian RJ Baccon J Knollmann-Ritschel B : The need for laboratory medicine in the Undergraduate Medical Education curriculum: a white paper from the association of pathology chairs. *Med Sci Educ.* 2023;34(1):193–200. 10.1007/s40670-023-01895-9 38510385 PMC10948729

[10] Nigatu T Deress T Mezgebu B : Determinants of quality laboratory service provision among government comprehensive specialized hospitals in Northwest Ethiopia. *Res Sq.* 2024. 10.21203/rs.3.rs-4789250/v1 PMC13169886 41923262

[11] Ishengoma DS Kamugisha ML Rutta ASM : Performance of health laboratories in provision of HIV diagnostic and supportive services in selected districts of Tanzania. *BMC Health Serv Res.* 2017;17(1): 70. 10.1186/s12913-017-2030-9 28114988 PMC5259978

[12] Kinyenje E Ngowi RR Msigwa YS : Status of countrywide laboratory services quality and capacity in primary healthcare facilities in Tanzania: findings from star rating assessment. *PLOS Glob Public Health.* 2023;3(10): e0001489. 10.1371/journal.pgph.0001489 37851603 PMC10584114

[13] Chidzaye RW : Assessing barriers to medical laboratory diagnostic service delivery in Mzuzu city. *Int J Biomed Sci.* 2019;15(1):32–56. 10.59566/IJBS.2019.15032

[14] O’Kane M : The reporting, classification and grading of quality failures in the medical laboratory. *Clin Chim Acta.* 2009;404(1):28–31. 10.1016/j.cca.2009.03.023 19302996

[15] Lubin IM Astles JR Shahangian S : Bringing the clinical laboratory into the strategy to advance diagnostic excellence. *Diagnosis (Berl).* 2021;8(3):281–294. 10.1515/dx-2020-0119 33554526 PMC8255320

[16] Adekoya A Okezue MA Menon K : Medical laboratories in healthcare delivery: a systematic review of their roles and impact. *Laboratories.* 2025;2(1):8. 10.3390/laboratories2010008

[17] Gandra S Alvarez-Uria G Turner P : Antimicrobial resistance surveillance in low- and middle-income countries: progress and challenges in eight South Asian and Southeast Asian countries. *Clin Microbiol Rev.* 2020;33(3):e00048–19. 10.1128/CMR.00048-19 32522747 PMC7289787

[18] Alemnji GA Zeh C Yao K : Strengthening national health laboratories in sub-Saharan Africa: a decade of remarkable progress. *Trop Med Int Health.* 2014;19(4):450–458. 10.1111/tmi.12269 24506521 PMC4826025

[19] Abera E Taye B Belay G : Factors Affecting Quality of Laboratory Services in Public and Private Health Facilities in Addis Ababa, Ethiopia. *EJIFCC.* 2017;28(3):205–223. 29075171 PMC5655637

[20] Dacombe R Squire S Ramsay A : Essential medical laboratory services: their role in delivering equitable health care in Malawi. *Malawi Med J.* 2007;18(2):77–79. 10.4314/mmj.v18i2.10914

[21] Khadambi-Morokane H Bhowan K Ayuk S : An overview of medical diagnostic laboratories in South Africa that meet the international standard of accreditation: ISO 15189. *The Journal of Medical Laboratory Science and Technology of South Africa.* 2021;3(1):27–34. 10.36303/JMLSTSA.2021.3.1.61

[22] Nkwawir SC Batumani NN Maruta T : From grass to grace: How SLMTA revolutionised the Bamenda Regional Hospital Laboratory in Cameroon. *Afr J Lab Med.* 2014;3(2):203. 10.4102/ajlm.v3i2.203 29043186 PMC5637803

[23] Beyanga M Gerwing-Adima L Jackson K : Implementation of the laboratory quality management system (ISO 15189): experience from Bugando Medical Centre Clinical Laboratory - Mwanza, Tanzania. *Afr J Lab Med.* 2018;7(1):657. 10.4102/ajlm.v7i1.657 30167386 PMC6111386

[24] Gumba H Waichungo J Lowe B : Implementing a quality management system using good clinical laboratory practice guidelines at KEMRI-CMR to support medical research [version 2; peer review: 2 approved]. *Wellcome Open Res.* 2019;3:137. 10.12688/wellcomeopenres.14860.2 30607370 PMC6305232

[25] Guzel O Guner EI : ISO 15189 accreditation: requirements for quality and competence of medical laboratories, experience of a laboratory I. *Clin Biochem.* 2009;42(4–5):274–278. 10.1016/j.clinbiochem.2008.09.011 19863920

[26] Zeh CE Inzaule SC Magero VO : Field experience in implementing ISO 15189 in Kisumu, Kenya. *Am J Clin Pathol.* 2010;134(3):410–418. 10.1309/AJCPZIRKDUS5LK2D 20716797

[27] Tanasiichuk I Karaman O Natrus L : Key success factors for the implementation of quality management systems in developing countries. *Afr J Lab Med.* 2023;12(1):2058. 10.4102/ajlm.v12i1.2058 36756216 PMC9900284

[28] Nzabahimana I Sebasirimu S Gatabazi JB : Innovative strategies for a successful SLMTA country programme: the Rwanda story. *Afr J Lab Med.* 2014;3(2):217. 10.4102/ajlm.v3i2.217 29043189 PMC5637798

[29] Moyo H Osawe S Nyangulu C : Hybrid mentorship of medical laboratories to achieve ISO 15189: 2012 accreditation in Malawi: The University of Maryland Malawi experience. *Glob Health Sci Pract.* 2024;12(6): e2400254. 10.9745/GHSP-D-24-00254 39547701 PMC11666087

[30] Mokobela KO Moatshe MT Modukanele M : Accelerating the spread of laboratory quality improvement efforts in Botswana. *Afr J Lab Med.* 2014;3(2):207. 10.4102/ajlm.v3i2.207 29043187 PMC5637812

[31] Musau SK Mwachari C Kirui E : Implementing an intensified mentorship approach towards accelerated medical laboratory accreditation in 10 counties in Kenya. *Afr J Lab Med.* 2022;11(1): 1814. 10.4102/ajlm.v11i1.1814 35937766 PMC9350484

[32] Nzombe P Luman ET Shumba E : Maximising mentorship: variations in laboratory mentorship models implemented in Zimbabwe. *Afr J Lab Med.* 2014;3(2):241. 10.4102/ajlm.v3i2.241 29043196 PMC5637805

[33] Ntshambiwa K Ntabe-Jagwer W Kefilwe C : Translating a national laboratory strategic plan into action through SLMTA in a district hospital laboratory in Botswana. *Afr J Lab Med.* 2014;3(2):209. 10.4102/ajlm.v3i2.209 29043188 PMC5637797

[34] Makokha EP Mwalili S Basiye FL : Using standard and institutional mentorship models to implement SLMTA in Kenya. *Afr J Lab Med.* 2014;3(2):220. 10.4102/ajlm.v3i2.220 29043191 PMC5637804

[35] Tembo D : Implementing QMS based on ISO15189 at MLW Laboratories_Data collected and Analysed. *figshare.* 2026. https://figshare.com/articles/dataset/Implementing_QMS_based_on_ISO15189_at_MLW_Laboratories_Data_collected_and_Analysed/30999691/1

