# Supplementary material for: Implementing and Transitioning a Laboratory Quality Management System from ISO 15189:2012 to ISO 15189:2022: Experience from the Malawi-Liverpool Wellcome Research Programme, Blantyre
Source: Wellcome Open Res. 2026 Apr 17;11:144. Originally published 2026 Feb 23. [Version 2] doi: 10.12688/wellcomeopenres.25356.2 (PMC13110388; doi:10.12688/wellcomeopenres.25356.2)
Supplement: Peer review history

# Reviewer report: Reviewer response for version 2

**DOI:** 10.21956/wellcomeopenres.28997.r153522

**Published:** 2026-04-25

**Authors:** Christoph Buchta

**Affiliations:** 1 Austrian Association for Quality Assurance and Standardization of Medical and Diagnostic Tests (ÖQUASTA), Vienna, Austria

Thank you for addressing all my comments. I agree with the changes you have made and have no further comments or recommendations. 

 The word that was thought to be missing from the text turned out to be a clerical error on my part, and I would like to apologise for this.

 Bext wishes!

Is the work clearly and accurately presented and does it cite the current literature?

Partly

If applicable, is the statistical analysis and its interpretation appropriate?

Partly

Are all the source data underlying the results available to ensure full reproducibility?

Yes

Is the study design appropriate and is the work technically sound?

Yes

Are the conclusions drawn adequately supported by the results?

Partly

Are sufficient details of methods and analysis provided to allow replication by others?

Yes

Reviewer Expertise:

General EQA methodology and use of EQA data for other than results assessment purposes; infection diagnostics, immuohematology; interpretation of ISO 15189 and compliance with requirements in this standard.

I confirm that I have read this submission and believe that I have an appropriate level of expertise to confirm that it is of an acceptable scientific standard.

# Reviewer report: Reviewer response for version 2

**DOI:** 10.21956/wellcomeopenres.28997.r153521

**Published:** 2026-04-20

**Authors:** Mercy A Okezue

**Affiliations:** 1 Michigan State University, East Lansing, MI, USA

The authors have revised the article in line with the suggestions provided for improvement.

Is the work clearly and accurately presented and does it cite the current literature?

Partly

If applicable, is the statistical analysis and its interpretation appropriate?

Partly

Are all the source data underlying the results available to ensure full reproducibility?

Yes

Is the study design appropriate and is the work technically sound?

Yes

Are the conclusions drawn adequately supported by the results?

Partly

Are sufficient details of methods and analysis provided to allow replication by others?

Yes

Reviewer Expertise:

Pharmaceutical Sciences, Regulatory Sciences, and Packaging Systems Sciences

I confirm that I have read this submission and believe that I have an appropriate level of expertise to confirm that it is of an acceptable scientific standard.

# Reviewer report: Reviewer response for version 1

**DOI:** 10.21956/wellcomeopenres.27940.r150295

**Published:** 2026-03-25

**Authors:** Christoph Buchta

**Affiliations:** 1 Austrian Association for Quality Assurance and Standardization of Medical and Diagnostic Tests (ÖQUASTA), Vienna, Austria

The authors report on the transition process from ISO 15189:2012 to ISO 15189:2022. This is an interesting report from a laboratory in Malawi.

 On the whole, the manuscript seems to be somewhat wordy; the long passages of text make it seem almost like an article for a weekend supplement’s arts section and thus distract from the key data and findings. These, however, are certainly worth indexing. Nevertheless, you can significantly shorten the length of the manuscript without losing any important information.

 To my opinion, everybody in the lab community knows ISO 15189 and it therefore needs no explanation in the Background section.

 The manuscript states on several occasions that ISO 15189:2022 is essential for clinical laboratories as well as for research laboratories; zhr secpnd claim, research laboratories, requires substantiation.

 A word is missing in the last sentence of the Conclusions paragraph in the Abstract.

 Method validation and verification is mentioned in a heading, but only on verification is reported. 

 Please do not switch between the terms ‘EQA’ and ‘PT’. Stick to one or the other.

 It would be interesting to know which clauses of the standard the non-conformities identified during the accreditation audit related to.

 I will be happy to review any revised version of the paper!

Is the work clearly and accurately presented and does it cite the current literature?

Partly

If applicable, is the statistical analysis and its interpretation appropriate?

Partly

Are all the source data underlying the results available to ensure full reproducibility?

Yes

Is the study design appropriate and is the work technically sound?

Yes

Are the conclusions drawn adequately supported by the results?

Partly

Are sufficient details of methods and analysis provided to allow replication by others?

Yes

Reviewer Expertise:

General EQA methodology and use of EQA data for other than results assessment purposes; infection diagnostics, immuohematology; interpretation of ISO 15189 and compliance with requirements in this standard.

I confirm that I have read this submission and believe that I have an appropriate level of expertise to confirm that it is of an acceptable scientific standard, however I have significant reservations, as outlined above.

# Response

**Published:** 2026-04-01

**Authors:** Dumizulu Tembo

**Affiliations:** Malawi Liverpool Wellcome Research Programme, Malawi

We thank the reviewer for their comment. They have been responded to as follows:

 On the whole, the manuscript seems to be somewhat wordy; the long passages of text make it seem almost like an article for a weekend supplement’s arts section and thus distract from the key data and findings. These, however, are certainly worth indexing. Nevertheless, you can significantly shorten the length of the manuscript without losing any important information.

*Response: Our intention was to provide sufficient context and narrative to highlight the practical relevance of the findings; however, we understand that some sections may appear overly wordy and could distract from the core results. We have reviewed the manuscript to remove redundant text, tighten explanations, and streamline the narrative to ensure that the key data and findings stand out more clearly.*  

 To my opinion, everybody in the lab community knows ISO 15189 and it therefore needs no explanation in the Background section.

*Response: We agree that ISO15189 is widely recognized among the technically experienced lab community. Our intention for this brief explanation was to ensure accessibility for all readers, including those who may not have prior exposure to accreditation standards. In our setting, for example significant number of trainees rotate through the lab encountering the ISO15189 for the first time.*  

 The manuscript states on several occasions that ISO 15189:2022 is essential for clinical laboratories as well as for research laboratories; zhr secpnd claim, research laboratories, requires substantiation.

*Response: The reviewer raises a good point. ISO15189 is designed for medical/clinical laboratories. Our intention was not to imply that ISO15189 is specifically mandatory for research labs but rather to say that for those research labs generating clinical data or supporting clinical studies, may wish to adopt these standard principles to strengthen quality.*  

 A word is missing in the last sentence of the Conclusions paragraph in the Abstract. *Response: The missing word could not be identified. If the reviewer could please clarify *  

 Method validation and verification is mentioned in a heading, but only on verification is reported.

*Response: This is acknowledged. The revised manuscript section heading reads Method Verification*  

 Please do not switch between the terms ‘EQA’ and ‘PT’. Stick to one or the other.

*Response: EQA is maintained throughout the revised manuscript*    

 It would be interesting to know which clauses of the standard the non-conformities identified during the accreditation audit related to.

*Response: We acknowledge the value of mapping non* *‑conformities to specific clauses of the standard. However, given the number of non* *‑conformities identified across the assessments, a detailed clause* *‑by* *‑clause listing for every individual finding would be repetitive and difficult to present succinctly. As a practical approach, we have listed the recommended areas for improvement for each assessment. This approach highlights gaps identified, while avoiding duplication, particularly where multiple findings relate to the same underlying clause.*

# Reviewer report: Reviewer response for version 1

**DOI:** 10.21956/wellcomeopenres.27940.r148885

**Published:** 2026-03-06

**Authors:** Mercy A Okezue

**Affiliations:** 1 Michigan State University, East Lansing, MI, USA

The authors demonstrated enhanced laboratory performance, improved documentation, and a stronger teamwork and culture of quality resulting from their institution’s efforts to implement and transition a QMS from the 2012 to the 2022 version of ISO 15189.

 The authors are advised to revise the manuscript, addressing the following observations:

**Introduction**

1. The main objective(s) of the paper were not clearly articulated in the first few paragraphs. Please revise this section to clearly state the importance of your study or what gap you are aiming to fill.
2. Here, and throughout the manuscript, please be more specific in the type of laboratory referenced in the 2 ^nd^ paragraph, …..”when considering essential health packages, quality laboratory services are indispensable”. For example, is this a medicine QC lab, medical diagnostic lab, etc.?

**Methodology**

 Clearly indicate the type of study presented in your manuscript

**Method validation and verification**

 This subtitle suggests a distinction between validation and verification. However, validation was not included in the narrative provided in this section. If none of the analytical methods used in your study required validation, I suggest you adjust the subtitle to reflect only verification.

**Discussions and Conclusion**

 In addition to the qualitative discussions of results, the authors are advised to also incorporate discussions of the quantitative data from the results obtained from the study.

Is the work clearly and accurately presented and does it cite the current literature?

Partly

If applicable, is the statistical analysis and its interpretation appropriate?

Partly

Are all the source data underlying the results available to ensure full reproducibility?

Yes

Is the study design appropriate and is the work technically sound?

Yes

Are the conclusions drawn adequately supported by the results?

Partly

Are sufficient details of methods and analysis provided to allow replication by others?

Yes

Reviewer Expertise:

Pharmaceutical Sciences, Regulatory Sciences, and Packaging Systems Sciences

I confirm that I have read this submission and believe that I have an appropriate level of expertise to confirm that it is of an acceptable scientific standard, however I have significant reservations, as outlined above.

# Response

**Published:** 2026-03-20

**Authors:** Dumizulu Tembo

**Affiliations:** Malawi Liverpool Wellcome Research Programme, Malawi

We sincerely thank the reviewer for their constructive feedback. All comments have been carefully addressed in the revised version as follows:

- A background paragraph has been added to clearly articulate the gap this study addresses and its aims.
- The type of laboratory involved has now been specified consistently throughout the manuscript.
- The Methods section has been updated to include a clear description of the study type. In addition, the term *validation* has been removed from the former “method validation and verification” subheading
- The Discussion section has also been modified in several areas to incorporate an expanded interpretation of the quantitative data.

We appreciate the reviewer’s time and valuable insights.
